# SNP rs2073618 of the Osteoprotegerin Gene Is Associated with Diabetic Retinopathy in Slovenian Patients with Type 2 Diabetes

**DOI:** 10.1155/2013/364073

**Published:** 2013-10-20

**Authors:** Sara Mankoč Ramuš, Tina Kumše, Mojca Globočnik Petrovič, Daniel Petrovič, Ines Cilenšek

**Affiliations:** ^1^Institute of Histology and Embryology, Medical Faculty, University Ljubljana, Korytkova 2, 1105 Ljubljana, Slovenia; ^2^University Medical Centre, Eye Clinic, Grablovičeva 46, 1000 Ljubljana, Slovenia

## Abstract

Recent studies indicate that osteoprotegerin (OPG) acts as an important regulatory molecule in the vasculature. Also, a strong association was observed between circulation OPG and microvascular complication. By considering the possible role of OPG in diabetic retinopathy (DR) we examined two of the most studied polymorphisms of the OPG genes rs2073618 (located in exon I) and rs3134069 (located in the promoter region) and their relation to DR in Slovenian patients with type 2 diabetes. Logistic regression analysis demonstrated that the carriers of the CC genotype had a 2.2 higher risk for DR than those with either the CG genotype or the GG genotype (codominant model for rs2073618). Furthermore, the combined effect of single nucleotide polymorphisms (SNPs) rs2073618 and rs3134069 on the DR was stronger than that of each SNP alone. The odds ratio (OR) for individuals with CC genotype (rs2073618) and AA genotype (rs3134069) compared with carriers of CG/GG (rs2073618) + AA (rs3134069) was 2.54 (95% CI = 1.26–5.13, *P* = 0.01). To conclude, these results indicate that SNPs in the OPG gene may be implicated in the pathogenesis of DR.

## 1. Introduction

Most diabetic patients, especially those with poor glycaemic control, develop diabetic retinopathy (DR), which remains the major cause of new-onset blindness among diabetic adults. DR is characterized by vascular permeability and increased tissue ischemia and angiogenesis [1]. It is known that for the development of DR both genetic and environmental factors are highly relevant [2]. DR is thought to be caused by oxidative stress, advanced glycation end-products (AGEs), inflammatory mediators, and endothelial cell death [3]. A member of the tumor necrosis factor (TNF) receptor superfamily glycoprotein osteoprotegerin (OPG), first identified in 1997, acts as an important regulatory molecule in the vasculature [4, 5]. It is expressed in the endothelial and smooth muscle cells, and it is modulated by proinflammatory cytokines and hormones, like insulin and TNF-*α* [6, 7]. OPG, also known as osteoclastogenesis inhibitory factor (OCIF), was originally discovered as an inhibitor of bone resorption [8]. This effect is due to binding and neutralisation of the receptor activator of the nuclear factor-*κ*B ligand, thereby neutralizing its functions and negatively regulating osteoclast differentiation, activity, and survival resorption [5, 8].

There have been several studies examining the associations between OPG polymorphisms and bone diseases [9–12], atherosclerosis [13–15], and macrovascular complications of diabetes [16–19]. On the contrary, not many studies have been performed to investigate the association between polymorphisms in the OPG gene and the risk for microvascular complications in type 2 diabetes [4, 6, 20, 21].

Therefore, the purpose of the present study was to examine whether there is a link between the rs2073618 (c.9C>G, G1181C) and rs3134069 (g.119964988A>C, T245G) polymorphisms of the OPG gene and DR in type 2 diabetic patients and to evaluate whether the combined effects of these gene variations influence the risk for DR.

## 2. Patients and Methods

In this cross-sectional case-control study 645 unrelated Caucasians with type 2 diabetes mellitus with a defined ophthalmologic status were enrolled (they have not been controlled for the glycemic history). Patients were classified as having type 2 diabetes according to the current American Diabetes Association criteria [22]. Fundus examination was performed by a senior ophthalmologist (M.P.) after pupil dilatation (tropicamide and phenylephrine 2.5%) using slit lamp biomicroscopy with noncontact lens and was electronically documented with a 50°-angle fundus camera (Topcon-TRC 40-IX; Tokyo, Japan). Staging of diabetic retinopathy was determined according to the ETDRS retinopathy severity scale [23]. The study group consisted of 645 subjects: 280 subjects with DR (cases) and the control group of 365 subjects with type 2 diabetes of more than 10 years' duration who had no clinical signs of DR.

To avoid the confounding effect of impaired kidney function, the patients with overt nephropathy were not enrolled. The study was approved by the national medical ethics committee. After an informed consent for the participation in the study was obtained, a detailed interview was made.

### 2.1. Genotyping

Genomic DNA was extracted from 200 *μ*L of whole blood using a FlexiGene DNA isolation kit according to the recommended protocol (Qiagene, Germany).

Based on the available literature, we chose two single nucleotide polymorphisms (SNPs): rs2073618 (located in exon I) and rs3134069 (located in the promoter region) of the OPG gene [24]. The G allele from rs2073618 results with aspartic acid substitution to lysine (N3 K) in the 3′ amino terminus in the OPG signal region which may lead to alterations in protein activity. SNPs were genotyped using the predesigned TaqMan SNP Genotyping Assays (Applied Biosystems, Foster City, CA, USA), C_1971047_1 and C_27464534_20, resp.). The reactions were performed using the StepOne system (Applied Biosystems, Foster City, CA, USA) according to the manufacturer's instruction. Real-time PCR reactions were set up in a final volume of 5 *μ*L containing 2.5 *μ*L of 2 × TaqMan Genotyping Master Mix (Applied Biosystems, Foster City, CA, USA), 0.12 *μ*L of 40 × SNP Genotyping Assay (Applied Biosystems, Foster City, CA, USA), 1.88 *μ*L of nuclease free water, and 25 ng of genomic DNA. PCR amplification was carried out under the following conditions: 10 min at 95°C enzyme activation followed by 55 cycles at 95°C for 15 s and at 60°C for 1 min.

### 2.2. Statistical Analysis

Statistical analyses were conducted with the use of the SPSS program for Windows version 19 (SPSS Inc. Illinois). Continuous clinical data were compared by unpaired Student's *t*-test, while chi-square test was used to compare discrete variables. Data were expressed as mean ± SD (continuous variables) or as the number and percent of patients (categorical variables). Further, all variables that showed significant differences by univariate analysis (with a *P* value < 0.05 considered significant) were analyzed together in a logistic regression analysis. A *P* < 0.05 was considered statistically significant. All *P* values were not adjusted for multiple testing. The deviation from Hardy-Weinberg equilibrium (HWE) was assessed by the exact test (http://ihg.gsf.de/) [25]. The linkage disequilibrium (LD) between the polymorphisms was quantified using Haploview version 4.2 (http://www.broad.mit.edu/mpg/haploview). Joint effects of both SNPs were analyzed in a logistic regression model, where different combinations between two genotype models were considered (recessive and dominant).

## 3. Results

The demographic and clinical data of 645 patients diagnosed with type 2 diabetes enrolled in the study are shown in Table 1. Among them, 365 patients had no evidence of DR (controls), and the remaining 280 had DR (cases). There were no significant differences between groups with respect to age, sex, systolic and diastolic blood pressure, history of hypertension, and smoking status. On the other hand, statistically significant difference was observed in the following parameters: duration of diabetes, insulin treatment, HbA_1c _, body mass index (BMI), and total LDL, HDL, and cholesterol levels. Cases with DR had more than 5 years of longer diabetes duration compared to the diabetics without DR. A significantly higher proportion of cases required insulin treatment in comparison to diabetics without DR. Cases had higher HbA_1c_, total cholesterol, and LDL cholesterol levels, whereas BMI and HDL cholesterol levels were statistically significantly lower than in controls.

As shown in Table 2, the frequencies of the CC (ancestral allele based on 1000 Genomes Project (1000 G) data) [26], CG, and GG genotypes of the rs2073618 polymorphism were 25.0%, 47.1%, and 27.9% in cases and 18.1%, 44.9%, and 37.0% in controls, respectively. The distributions of the CC, AC, and AA (ancestral allele based on 1000 G data) genotypes of the rs3134069 polymorphism were 2.2%, 17.1%, and 80.7% in cases and 0.3%, 9.9%, and 89.8% in controls, respectively. The average frequency of the ancestral alleles in the whole study population (the C allele for rs2073618 and the A allele for rs3134069) was 44% for C and 92.4% for A, which was slightly lower than that observed in Caucasians according to the 1000 G data. No significant differences in the allele frequencies were found, when the merged data of Slovenian patients with type 2 diabetes (patients with diagnosed DR and those without DR) were compared with 156 healthy individuals data for both SNPs (data not shown). Ancestral allele frequencies for each OPG polymorphism (rs2073618 and rs3134069) in the group of healthy individuals were as follows: 43.6% for C and 93.3% for A, respectively.

Both SNPs conformed to HWE in both the case (rs2073618, *P* = 0.35; rs3134069, *P* = 0.08) and control (rs2073618, *P* = 0.19; rs3134069, *P* = 0.99) group. Although there was a moderate LD between analysed SNPs (D′= 0.71), SNPs cannot substitute each other because rs2073618 and rs3134069 have a very low correlation (*r* ^2^ = 0.05) with each other.

It was revealed that genotype and allele distribution of both SNPs differed significantly between cases and controls. The C allele and the CC genotype of the rs2073618 were significantly more frequent in cases (*P* = 0.004; *P* = 0.002). Next, the frequency of the A allele and AA genotype was significantly less frequent in cases than in the control group (*P* = 0.0002; *P* = 0.001) (Table 2).

Following these observations, we used a logistic regression analysis to evaluate whether these SNPs were independently associated with DR after adjusting for duration of diabetes, insulin therapy, BMI, HbA_1c_, total LDL, HDL, and cholesterol levels. The CC genotype of the rs2073618 polymorphism was significantly associated with the increased risk for DR compared with the GG genotype (OR_Co-dom_ = 2.19, 95% CI = 1.18–4.09, *P* = 0.01) in the codominant model. The association of the rs2073618 with DR was also obtained when applying the dominant model (OR = 1.72, 95% CI = 1.08–2.70, *P* = 0.02). Using the CG and GG genotypes combined as reference in the recessive model, the OR for the CC genotype was 1.75 (95% CI = 1.0–3.05, *P* = 0.05). On the other hand, the association of rs3134096 with DR was not significant in dominant and recessive models (Table 3).

**Figure 1:**
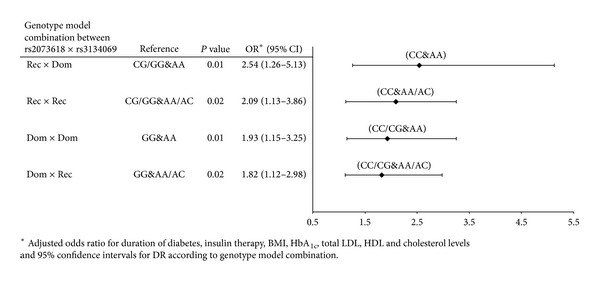
Joint effect of rs2073618 and rs3134069 on the genetic risk for DR.

The final step of our study was to evaluate whether the combined effects of these SNPs influence the risk for DR. We used logistic regression to investigate the joint effect of the two SNPs. The results indicate a significant interaction effect between these two SNPs as risk factors for DR, after adjusting for confounding variables found relevant in univariate analysis, rendering an OR_Rec×Dom⁡_ of 2.54 (95% CI = 1.26–5.13, *P* = 0.01) for individuals with CC genotype (rs2073618) and AA genotype (rs3134069) compared with carriers of CG/GG (rs2073618) + AA (rs3134069). In addition, carriers of CC (rs2073618) + AA/AC (rs3134069) had a significantly increased risk for DR (OR_Rec×Rec_ = 2.09, 95% CI = 1.13–3.86, *P* = 0.02) compared with carriers of CG/GG (rs2073618) + AA/AC (rs3134069). Moreover, carriers of CC/CG (rs2073618) + AA (rs3134069) had a significantly increased risk for DR (OR_Dom⁡×Dom⁡_ = 1.93, 95% CI = 1.15–3.25, *P* = 0.01) compared with carriers of GG (rs2073618) + AA (rs3134069). Finally, carriers of CC/CG (rs2073618) + AA/CA (rs3134069) had OR_Dom⁡×Rec_ of 1.82 (95% CI = 1.12–2.98, *P* = 0.02) relative to carriers of GG (rs2073618) + AA/AC (rs3134069) (Figure 1).

## 4. Discussion

To the best of our knowledge, this is the first study to demonstrate an association between SNP rs2073618 of the OPG gene and DR in Caucasians with type 2 diabetes. As for the aforementioned SNP we proved that the minor C allele (*P* = 0.004) occurred more frequently in diabetic patients with DR. Logistic regression analysis demonstrated that the carriers of the CC genotype had a 2.2 higher risk for DR than those with either the CG genotype or the GG genotype (codominant model). Furthermore, in a dominant model, the carriers of at least one C allele (CG + CC genotypes) were found to modify susceptibility for DR. The occurrence of DR was 1.71-fold higher in carriers with the C allele.

Likewise, Biscetti et al. also reported an independent association between the CC genotype and ischemic stroke in Italian diabetic patients. The higher-risk genotype conferred a 3.03-fold increased risk [27]. Moreover, in another study on patients with type 2 diabetes, a strong association was observed between the C allele and diabetic foot. Patients with CC genotype had a 1.72 increased risk for diabetic foot [28].

Although the second SNP rs3134069 was not associated with DR in the present study, the inclusion of the AA genotype in the Rec×Dom (rs2073618 × rs3134069) model further increased the risk for DR, rendering an OR_Rec×Dom⁡_ of 2.54. Furthermore, the combination of both SNPs (rs2073618 × rs3134069) in the following models: Rec×Rec, Dom×Dom, and Dom×Rec conferred a significantly increased risk for DR. Interestingly, in a Polish study, the AA genotype at the SNP rs3134069 was associated with an independently and significantly increased risk for the Charcot neuroarthropathy, with an OR of 11.5, compared to the diabetics with AC or CC genotypes [29].

Intriguingly, our study indicates that the combination of rs2073618 × rs3134069 co-ordinately might have a greater effect on the susceptibility for DR than revealed by the individual SNPs. Thus, suggesting a possibility that both SNPs are in linkage disequilibrium with other still unknown SNPs of the OPG gene or other genes having an effect on OPG expression, secretion, structure, or action.

Although it is known that the OPG gene variants are functionally important, the pathogenetic mechanism of the OPG gene variants in the development of DR remains unclear [24, 27, 29]. Variations in exon 1 of the OPG gene could result in a qualitative alteration of OPG synthesis, thus compromising its function as a decoy receptor, whereas variations in the sequence of the promoter region of the OPG gene could result in altered binding of different transcription factors, thus affecting the expression of OPG [24].

Elevated serum concentrations of OPG are found in a range of cardiovascular pathologies, suggesting the potential value of OPG as a biomarker of vascular risk and prognosis [30]. Also, in a study on diabetic people, a strong association was observed between circulating OPG and microvascular complications [4, 21, 31]. It is suggested that insulin resistance and inflammatory cytokines might mediate upregulation of the OPG release observed in humans and may reflect the endothelial dysfunction in subjects with diabetes [32]. One of inflammatory factors that are elevated in the early stages of DR is TNF-*α* [33]. The possible role of the OPG in the pathology of DR may relate to OPG production from vascular cells, since OPG synthesis is regulated by TNF-*α* in endothelial cells [34]. There is a lot of evidence that endothelial dysfunction is closely connected to the development of DR [35]. Despite few attempts have been made to elucidate the relation between OPG and endothelial dysfunction [4, 18, 32], we are still far from a comprehensive understanding.

Other limitations of our study, such as the lack of direct biochemical evidence indicating the correlation of gene polymorphisms with altered expression of the OPG gene, small sample size, and cross-sectional design, suggest that further studies, preferably prospective in nature, are needed to elucidate the role of the OPG polymorphisms involved in the DR development.

To conclude, in Slovenian population an association between the SNPs combination rs2073618 × rs3134069 and DR was found. To the best of our knowledge no such SNP × SNP interactions have been found in patients with DR so far. In addition, this is the first study to implicate the CC genotype and hence the C allele, as the genetic risk factors for DR in Caucasians. These results indicate that SNPs in the OPG gene may be implicated in the pathogenesis of DR. However, further functional and biological evidence would be needed to confirm the suggestive influence of OPG polymorphisms on DR.

## Figures and Tables

**Table 1:** Clinical and laboratory characteristics of cases and controls.

Characteristics	Cases	Controls	*P* value
Number	280	365	
Age (years)	64.4 ± 9.5	63.9 ± 9.8	0.6
Male sex (%)	146 (52.2)	187 (51.2)	0.8
Duration of diabetes (years)	18.3 ± 8.1	12.5 ± 2.1	**<0.001**
Patients on insulin therapy (%)	195 (69.8)	148 (40.5)	**<0.001**
HbA_1c_ (%)*	8.0 ± 1.5	7.7 ± 1.4	**0.009**
Systolic blood pressure (mm Hg)	146 ± 22	144 ± 19	0.2
Diastolic blood pressure (mm Hg)	84 ± 11	84 ± 10	0.9
BMI (kg/m^2^)	28.8 ± 4.9	31.3 ± 4.4	**0.007**
History of hypertension (%)	218 (77.7)	285 (78)	0.5
Smokers (%)	28 (9.9)	24 (6.7)	0.2
Total cholesterol (mmol/L)	5.1 ± 1.2	4.7 ± 1.2	**0.005**
HDL cholesterol (mmol/L)	1.1 ± 0.3	1.2 ± 0.3	**0.007**
LDL cholesterol (mmol/L)	3.0 ± 1.0	2.7 ± 0.9	**0.001**
Triglycerides (mmol/L)	2.4 ± 1.9	2.2 ± 1.6	0.4

The values represent mean ± standard deviation. Bold indicates statistically significant results.

*The average value for haemoglobin A_1c_ (HbA_1c_).

**Table 2:** Distribution of rs2073618 and rs3134069 genotypes and alleles in patients with diabetic retinopathy (cases) and in those without diabetic retinopathy (controls).

	Cases (280)	Controls (365)	*P* value
rs2073618 (c.9C>G)			
CC	70 (25)	66 (18.1)	
CG	132 (47.1)	164 (44.9)	**0.02**
GG	78 (27.9)	135 (37)	
C allele (%)	272 (48.6)	296 (40.5)	
G allele (%)	288 (51.4)	434 (59.5)	**0.004**

rs3134069 (g.119964988A>C)			
CC	6 (2.2)	1 (0.3)	
AC	48 (17.1)	36 (9.9)	**0.001**
AA	226 (80.7)	328 (89.8)	
C allele (%)	60 (10.7)	38 (5.2)	
A allele (%)	500 (89.3)	692 (94.8)	**0.0002**

Bold indicates statistically significant results.

**Table 3:** Association between OPG polymorphisms and the risk for DR.

Polymorphism	Model	Number of cases/controls	OR* (95% CI)	*P* value
rs2073618 (c.9C>G)	Codominant:			
	CC versus GG (reference)	70/66 versus 78/135	2.19 (1.18–4.09)	**0.01**
	CG versus GG (reference)	132/164 versus 78/135	1.51 (0.93–2.47)	0.098
	Dominant:			
	CC + CG versus GG (reference)	202/230 versus 78/135	1.72 (1.08–2.70)	**0.022**
	Recessive:			
	CC versus CG + GG (reference)	70/66 versus 210/299	1.75 (1.0–3.05)	0.05

rs3134069 (g.119964988A>C)	Dominant:			
	CC + AC versus AA (reference)	54/37 versus 226/328	1.25 (0.67–2.33)	0.491
	Recessive:			
	CC versus AC + AA (reference)	6/1 versus 274/364	4.8 (0.45–50.8)	0.19

*Adjusted for duration of diabetes, insulin therapy, BMI, HbA_1c_, total LDL, HDL, and cholesterol levels.

Bold indicates statistically significant results.
